# Practical Notes on Diagnosis and Treatment

**Published:** 1908-02-15

**Authors:** 


### Practical Notes on Diagnosis and Treatment.

**Status Lymphaticus.**

This condition shows a distinct tendency to improve with advancing years, and where it has caused no serious organic lesions the subject will feel stronger and enjoy better health during the second, than he has during the first part of life.—*Dr. Louis Vintras.*

**Incontinence of Fæces.**

This is a less common condition in children than is incontinence of urine, but is much more easily checked. The most effectual treatment is the administration of Dover's power in doses of 1½ to 3 grains, according to the age of the child, three times a day, and a mixture of belladonna with potassium bromide, to which arsenic and nux vomica may be usefully added in some cases.—*Dr. Still.*

**Calcium Salts in Pneumonia.**

Calcium is a valuable drug in the hæmorrhagic type of pneumonia, but in the dry type of cases it is more rational to prescribe citrate of sodium or potassium, owing to their power of increasing the alkalinity as well as the fluidity of the blood.—*Dr. Douglas Cree.*

**Salicylates in Rheumatism.**

Dr. Lees considers that sodium salicylate is often given in very inadequate doses. He advocates increase of the dose until the maximum is reached, and this in an adult may mean 450 or more grains in 24 hours. With the salicylate he orders double doses of sodium bicarbonate. Unless this is done there is risk of acid intoxication and of a condition resembling diabetic coma.

